# Correction to: ‘Multiple paternity and number of offspring in mammals’ 2018 by Dobson *et al.*

**DOI:** 10.1098/rspb.2022.1957

**Published:** 2022-10-26

**Authors:** F. Stephen Dobson

*Proc. R. Soc. B* **285**, 20182042. (Published online 14 November 2018). (https://doi.org/10.1098/rspb.2018.2042)

This article corrects the following:

In ‘Multiple paternity and number of offspring in mammals’, a typographical error appeared in equation (2.2):

p=P(S>1|k,q)=1−P(S=1|k,q)

$$=\;\frac{1\;-\;kq{\left(1-q\right)}^{k-1}}{1-{\left(1-q\right)}^{k}}$$

which must be replaced by:

$$\begin{array}{rl} \;p & =P(S>1|k,q)=1-P(S=1|k,q)\\ & =1-\frac{kq{\left(1-q\right)}^{k-1}}{1-{\left(1-q\right)}^{k}} \end{array}$$

This error resulted in minor changes to results, but not to the discussion of the results or overall conclusions of the article.

Figure 1*a*, which currently shows estimates for $p_{B}$ ranging from 0.619 to 0.892 with a mean of 0.737:

**Figure:**
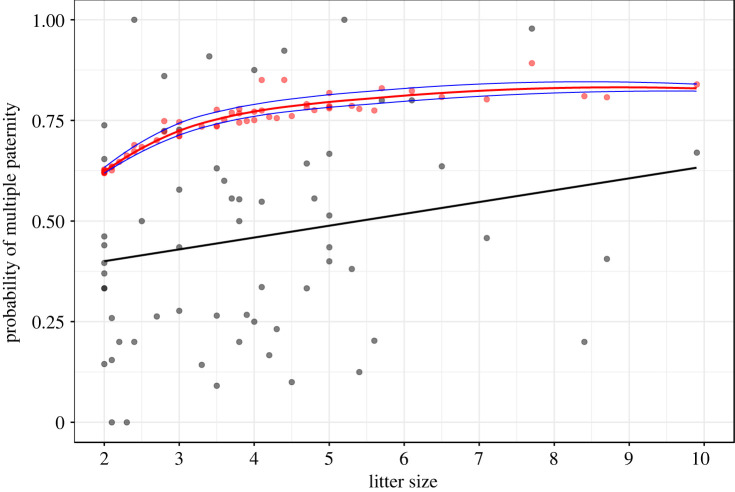

should instead show estimates for $p_{B}$ ranging from 0.392 to 0.860 with a mean $p_{B}$ of 0.594:

**Figure:**
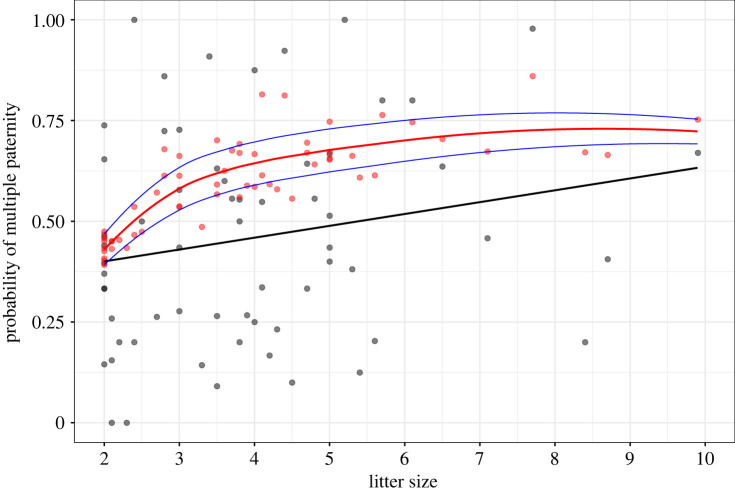

Similarly, figure 3 currently shows $p_{B}-p$ to range between –0.33 and 0.68 with a mean of 0.29 for mammalian species:

**Figure:**
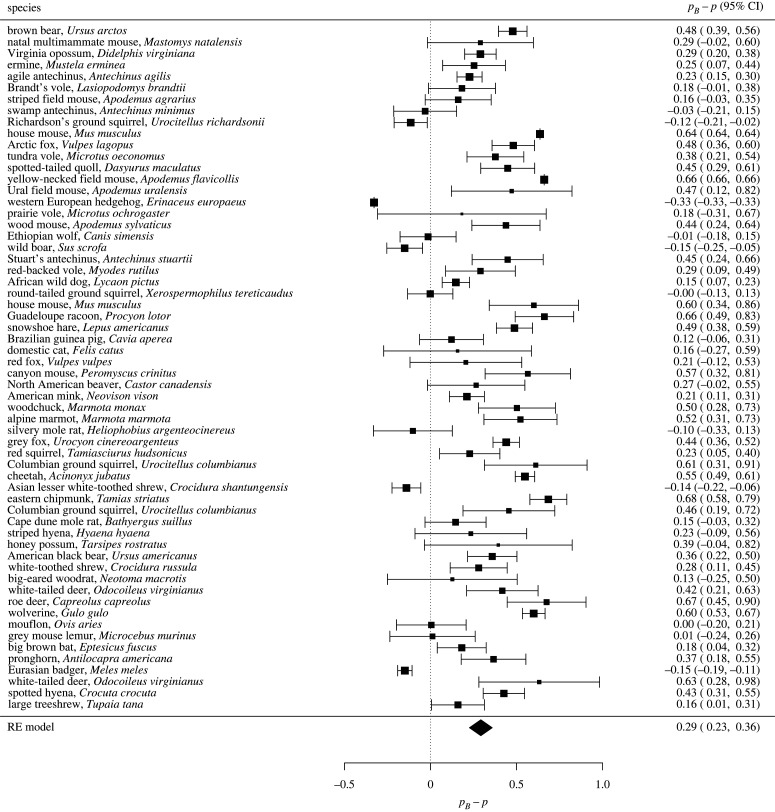

but instead should show values of $p_{B}-p$ to range from –0.49 to 0.56 with a mean of 0.15:

**Figure:**
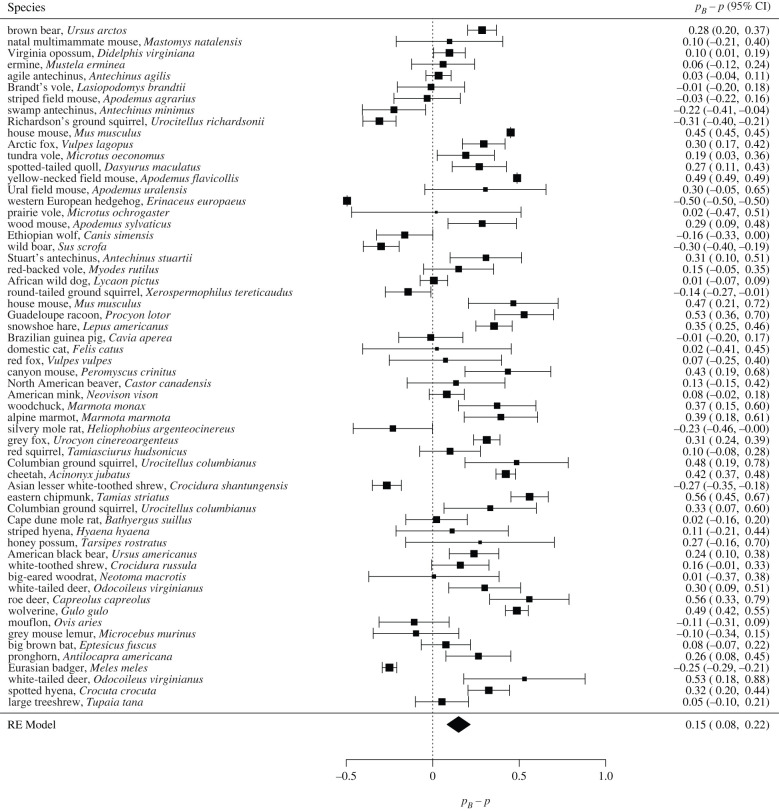

Results referencing figure 3 that stated:

‘Differences between the Bayesian estimates and actual values of the proportion of multiple paternity were significantly positive for 62% of the observed populations (figure 3), and the mean difference was also significantly positive (0.293 ± 0.034 s.e., *p* < 0.001, *N* = 60). Litter size had a non-significant impact on the mean difference (0.003 ± 0.019, *p* = 0.861, *N* = 60).’

should instead read:

‘Differences between the Bayesian estimates and actual values of the proportion of multiple paternity were significantly positive for 45% of the observed populations (figure 3), and the mean difference was also significantly positive (0.150 ± 0.034 s.e., *p* < 0.001, *N* = 60). Litter size had a non-significant impact on the mean difference (0.019 ± 0.018, *p* = 0.292, *N* = 60)’.

We have updated the code and provided the complete dataset on the Dryad Digital Repository at: https://doi.org/10.5061/dryad.d6kj502 [1].
